# Longitudinal analysis of hospital morbidity and mortality due to skin cancer from 2012 to 2022: comparison with demographic and geographic factors in Brazilian states^☆^

**DOI:** 10.1016/j.abd.2024.05.008

**Published:** 2024-12-31

**Authors:** Lucía Alejandra Bolis Castro, Maria Cecília Aires Martins, Kelser de Souza Kock

**Affiliations:** Department of Medicine, Universidade do Sul de Santa Catarina, Tubarão, SC, Brazil

**Keywords:** Ethnic distribution, Geography, Indicators of morbidity and mortality, Melanoma, Skin, Skin neoplasms

## Abstract

**Background:**

Skin cancer is the most common neoplasm among Brazilians, accounting for 31.3% of all cancer cases in the country.

**Objectives:**

To analyze the longitudinal morbimortality of skin cancer and compare it with the latitude and the prevalence of white skin color in each federative unit of the country.

**Methods:**

An ecological observational study was carried out using data from the Hospital Information System (SIH) and the Mortality Information System (SIM).

**Results:**

In Brazil, morbidity rates (per 100,000 inhabitants) increased from 14.88 to 27.69 (p < 0.05) when comparing the years 2012 and 2022, whereas the mortality rates (per 100,000 inhabitants) increased from 0.35 to 0.54 (p < 0.05) during the same period. Furthermore, most states showed an increase in morbimortality rates. Concerning race/ethnicity, individuals with white skin had the highest morbimortality rates. Regarding the age groups, individuals over 50 years old had the highest morbidity rates, while patients over 80 years old had the highest mortality rates, at 23.29%. Finally, the latitude showed a negative correlation, meaning that lower latitudes presented higher rates of morbidity and mortality from skin cancer.

**Study limitations:**

Self-reporting of skin color by participants, which may not fully correspond to phototypes. Data was analyzed exclusively from hospitalizations through the Unified Health System (SUS) and severe cases of the disease, as this study focused on hospitalizations and mortality, considering that the main treatment for skin cancer is carried out on an outpatient basis.

**Conclusions:**

The majority of Brazilian states showed rising morbimortality rates during the period, with the white-skinned population being the most affected. Additionally, a negative correlation with latitude was observed.

## Introduction

The most common neoplasm among Brazilians is skin cancer, accounting for 31.3% of all cancer cases in the country, whereas breast cancer is the second most common (10.5%), followed by prostate cancer (10.2%), colon and rectum (6.5%), lung (4.6%) and stomach cancer (3.1%).^1^ Anyone can develop skin cancer; however, the main risk factors are: light skin color, light-colored eyes, albinos and a personal or family history of this type of cancer, as well as patients undergoing treatment with immunosuppressants, those with previous skin diseases, prolonged and cumulative exposure to the sun, in addition to the use of tanning beds. Furthermore, due to intense sun exposure among young people, the incidence of skin cancer is becoming more frequent in age groups under 40. Although historically more common in older people, the trend is changing due to early exposure to the sun.^2^, ^3^

This tumor can be divided into melanoma skin cancer (MSC) and non-melanoma skin cancer (NMSC). Non-melanomas are the most common, accounting for more than 176,000 cases per year in Brazil. Their main types are basal cell carcinoma and squamous cell carcinoma, which generally have a lower mortality rate due to their slow growth and local invasion. Basal cell carcinoma, responsible for 80% of NMSC cases, generally has a less aggressive course compared to squamous cell carcinoma, which grows faster and can spread to other areas of the body. Therefore, when detected early, the prognosis tends to be favorable.^4^

Although skin cancer is the most common in the country, MSC is the least common, representing only 3% of neoplasms. However, it is the most serious, due to its possibility of causing metastasis with high mortality. In the last decade, due to the introduction of new medications and early detection of lesions, there has been an improvement in the survival of patients with melanoma. Thus, if detected in its early stages, it generally has a good prognosis.^5^

Thus, early diagnosis increases the chances of successful treatment, allowing less invasive interventions and preserving patients quality of life. From this perspective, it is essential that each patient recognize his own skin pattern, as well as identify risk phototypes, aiming at both primary and secondary prevention.^3^ According to the Fitzpatrick classification, individuals with phototypes I and II are more likely to develop malignant skin neoplasia.^6^, ^7^, ^8^

The profile of categories I and II are people with white skin, light colored hair, and light colored eyes, who burn easily when exposed to the sun and rarely/never tan.^8^ These attributes of each patient are inherited. Skin color, for instance, is related to the constitutive pigmentation of the skin, which is genetically inherited. Thus, heredity plays an important role in the development of melanoma.^9^

In Brazil, the southern region has a prevalence of white population of 75.1% and in the southeastern region of 50.7%.^10^ As a result, it is worth noting that these regions have the higher incidence of skin cancer cases in the country. According to data from the Brazilian Society of Dermatology (SBD, *Sociedade Brasileira de Dermatologia*), in approximately one decade (2008–2017) 33,000 Brazilians died due to skin cancer and the mortality rate was higher in the southeastern and southern regions of Brazil.^11^

The latitude of the location also contributes significantly to the incidence of skin cancer. It is known that countries closer to the equator (low latitude) have a higher annual incidence of sunlight. Thus, they have more intense UVB exposure than other regions. However, their exposure is continuous, unlike the sun exposure that occurs in high latitude regions, which have intermittent sunlight, resulting in a risk factor for the development of skin cancer.^11^

Also in relation to understanding the latitude of Brazil, it would be expected that the northeastern and midwestern regions would show higher records of cases of skin cancer.^11^ However, the reduction of the stratospheric ozone layer near the south pole contributes to a greater amount of UV rays that are harmful to human health,^12^ consequently contributing to the higher incidence of skin cancer both in the south of Brazil and in Australia and New Zealand.^13^

Considering that it is essential to understand the epidemiology of these neoplasms in Brazil, the aim of this study was to longitudinally analyze the morbidity and mortality of skin cancer in the period from 2012 to 2022 and compare it with the latitude and prevalence of white skin color in each federative unit of the country.

## Method

An ecological, time-series study was carried out, using as database the cases of hospital morbidity and mortality from skin cancer in the period from 2012 to 2022, compared with data on latitude (capitals) and prevalence of white skin color.

The records on hospital morbidity and mortality from skin neoplasms (ICD C43 and C44) were analyzed and collected from the DATASUS database of the Ministry of Health, through the TABNET application,^14^ using the variables in the period from 2012 to 2022 in the Brazilian states.

Morbidity and mortality rates for skin cancer were calculated by the ratio between the frequency of hospitalizations and deaths and the estimated population for each year and state of residence, the result of which was multiplied by the constant 100,000 (inhabitants), according to the formulas below:

*Hospital morbidity rate* = (*Number of hospitalizations in each year, age group and Brazilian state/estimated population for each year, age group and macro-region*) × 100,000.

*Mortality rate* = (*Number of deaths recorded each year, age group and macro-region/estimated population for each year, age group and Brazilian state*) × 100,000.

Population data came from 2010 census data and intercensal estimates for the other years, provided by the Brazilian Institute of Geography and Statistics (IBGE, *Instituto Brasileiro de Geografia e Estatística*)^15^ and mirrored by DATASUS.

Data on the distribution of morbidity and mortality due to skin cancer were collected regarding the variables of gender, age group and skin color during the entire study period, from 2012 to 2022, in the Brazilian states. Among the exclusion criteria, hospitalizations of non-residents in the Brazilian states where they were admitted to hospital units and cases with missing data (ignored or unavailable data) in the variables selected for the study were considered.

The dependent study variables were hospitalization and mortality due to “malignant skin neoplasm” (C43) and “other malignant skin neoplasms” (C44) and Brazilian federative units of residence (Acre, Alagoas, Amapá, Amazonas, Bahia, Ceará, Distrito Federal, Espírito Santo, Goiás, Maranhão, Mato Grosso, Mato Grosso do Sul, Minas Gerais, Pará, Paraíba, Paraná, Pernambuco, Piauí, Rio de Janeiro, Rio Grande do Norte, Rio Grande do Sul, Rondônia, Roraima, Santa Catarina, São Paulo, Sergipe and Tocantins). The latitude of the capital of the respective state and the prevalence of white skin color (IBGE) were considered independent variables.^15^

As the proposed study was of the ecological type, the database used as the data source is of public domain and access and does not contain information about the identity of participants or any personal information that allows individual identification or puts the confidentiality of the data at risk. Based on the above, and in accordance with the provisions of Resolution 510/2016 of the National Health Council (CNS, *Conselho Nacional de Saúde*), Article 1, Exclusive Paragraph, Subparagraphs II, III and V, this project does not fall within the terms of CNS Resolution 466/2012 for registration and analysis by Ethics Committees in Research Involving Human Beings.

The data were organized and stored using Microsoft Excel software and analyzed using the SPSS Software Version 20.0, Chicago: SPSS Inc; 2009. The quantitative variables were described using measures of central tendency and data dispersion. The qualitative variables were described using absolute and percentage frequencies. Linear regression was performed to analyze the temporal trend of skin cancer morbidity and mortality in Brazilian states and correlate it with the prevalence of white skin color and latitude (Brazilian capitals). The level of statistical significance was set at 5% (p-value < 0.05).

## Results

According to data from DATASUS, from 2012 to 2022, there were 483,228 hospitalizations due to malignant skin neoplasms in Brazil. Moreover, morbidity rates (×100,000/inhabitants) increased from 14.88 to 27.69 (p < 0.05) when comparing the years 2012 and 2022. Among the Federative Units that showed an increase in their rates are: Roraima, Pará, Tocantins, Maranhão, Ceará, Rio Grande do Norte, Alagoas, Sergipe, Bahia, Minas Gerais, Espírito Santo, Rio de Janeiro, Paraná, Santa Catarina, Rio Grande do Sul, Mato Grosso do Sul and the Federal District. Only the state of Piauí had a decreasing rate. The other states maintained stable values​​. Regarding mortality rates, when comparing the years 2012 and 2022, there was an increase in Brazil from 0.35 to 0.54 (with statistical significance, p < 0.05). Among the states that recorded an increase in their rates, the following stand out Pará, Maranhão, Ceará, Rio Grande do Norte, Paraíba, Pernambuco, Sergipe, Minas Gerais, São Paulo, Paraná, Santa Catarina and Goiás. The other states maintained stable rates (Table 1).

**Table 1:** Hospitalization and mortality rates due to malignant skin neoplasms (×100,000/inhab.) according to the federative unit and year of occurrence. Brazil, 2012 and 2022.

FU	Morbidity rates			Mortality rates		
	2012	2022		2012	2022	
RO	2.08	15.3	Increasing^a^	0.24	0.65	Stability
AC	3.79	3.26	Stability	0.38	0.22	Stability
AM	1.52	1.52	Stability	0.11	0.16	Stability
RR	2.71	7.85	Stability	0.62	0	Stability
PA	1.78	3.75	Increasing^a^	0.11	0.35	Increasing^a^
AP	1.79	2.23	Stability	0.14	0.11	Stability
TO	10.21	18.14	Increasing^a^	0.14	0.18	Stability
MA	7.24	13.47	Increasing^a^	0.07	0.31	Increasing^a^
PI	15.93	6.76	Decreasing^a^	0.34	0.27	Stability
CE	6.56	25.81	Increasing^a^	0.13	0.23	Increasing^a^
RN	22.07	49.54	Increasing^a^	0.15	0.31	Increasing^a^
PB	4.1	14.31	Stability	0.18	0.37	Increasing^a^
PE	18.93	17.43	Stability	0.23	0.52	Increasing^a^
AL	7.4	25.47	Increasing^a^	0.19	0.15	Stability
SE	3.25	11.14	Increasing^a^	0.14	0.25	Increasing^a^
BA	8.95	16.89	Increasing^a^	0.27	0.33	Stability
MG	10.65	22.82	Increasing^a^	0.31	0.5	Increasing^a^
ES	16.26	37.04	Increasing^a^	0.35	0.55	Stability
RJ	6.06	12.76	Increasing^a^	0.62	0.68	Stability
SP	20.82	29.22	Stability	0.44	0.58	Increasing^a^
PR	39.89	81.85	Increasing^a^	0.54	1.06	Increasing^a^
SC	21.53	63.95	Increasing^a^	0.72	1.29	Increasing^a^
RS	25.69	55.88	Increasing^a^	0.51	0.89	Increasing^a^
MS	15.84	40.8	Increasing^a^	0.31	0.59	Stability
MT	13.34	20.15	Stability	0.22	0.55	Stability
GO	16.04	22.51	Stability	0.11	0.4	Increasing^a^
DF	5.73	12.49	Increasing^a^	0.4	0.29	Stability
Brazil	14.88	27.69	Increasing^a^	0.35	0.54	Increasing^a^

Technical Notes: ^a^ p < 0.05 (statistical significance).

**Fig. 1:**
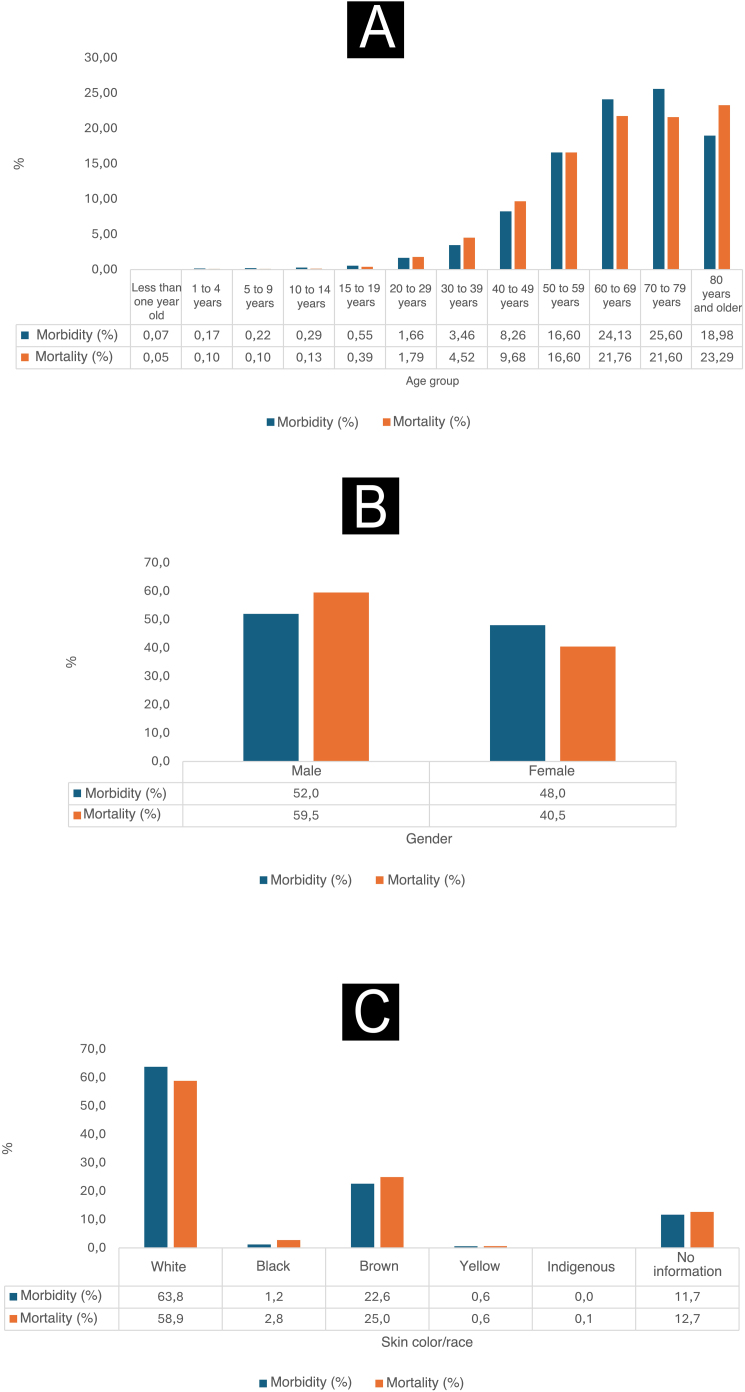
Hospitalization and mortality rates due to malignant skin neoplasms (×100,000/inhabitants) according to age group (A), gender (B) and self-reported skin color (C). Brazil, 2012‒2022. Source: SIH and SIM, adapted by the author.

As shown in Fig. 1, between 2012 and 2022, it is observed that the proportions of morbidity and mortality related to malignant skin neoplasms were higher among individuals with self-reported white skin color. The morbidity percentage reached 63.8%, while the mortality percentage was 58.9% for this specific population. Moreover, during this period, men had the highest proportions of morbidity, which reached 52%, and the highest mortality, reaching 59.5%. When analyzing the age groups, it was observed that the groups 50 to 59 years old, 60 to 69 years old and 70 to 79 years old were the most affected by malignant skin neoplasms in terms of morbidity, with percentages of 16.6%, 24.13% and 25.6%, respectively. On the other hand, the group over 80 years old had higher mortality rates, at 23.29%, while the mortality rates for the groups aged 50 to 59 years, 60 to 69 years and 70 to 79 years were 16.6%, 21.76% and 21.6%, respectively.

**Fig. 2:**
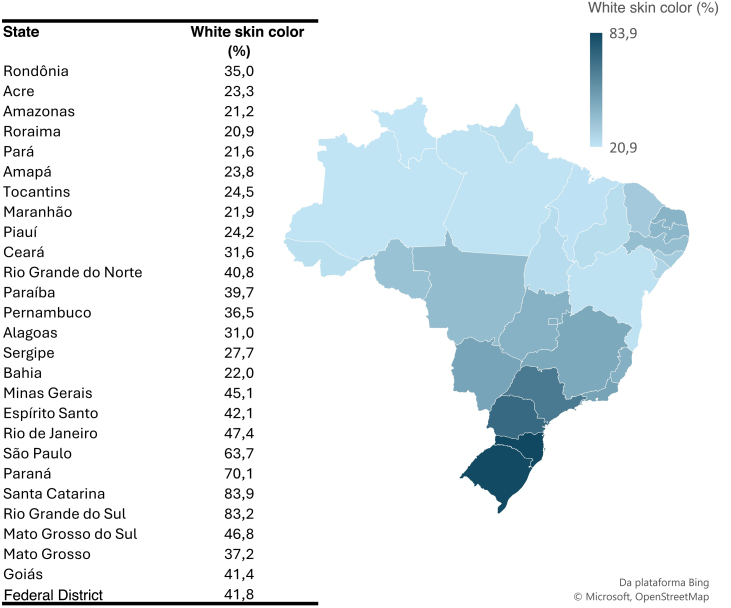
Prevalence (%) of white skin colored people in the Brazilian states. Source: SIH and SIM, adapted by the author.

Fig. 2 shows that the state of Santa Catarina had a higher prevalence of white skin colored individuals, reaching 83.9%, while Roraima had a lower proportion of this same population, with only 20.9%.

**Fig. 3:**
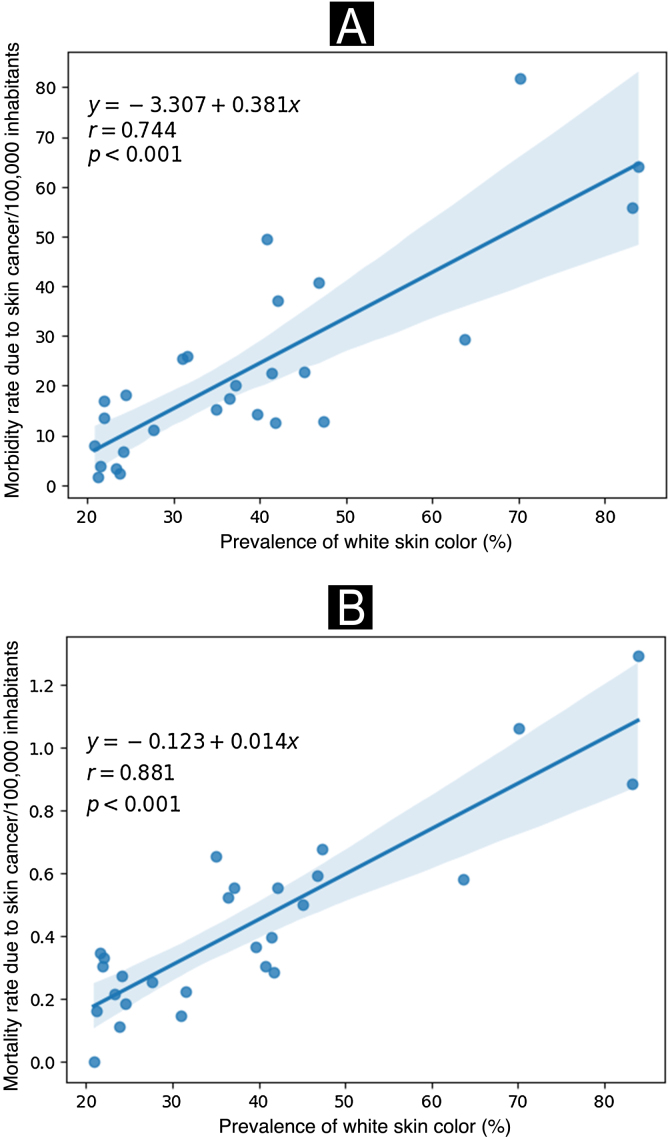
Hospitalization (A) and mortality rates (B) due to malignant skin neoplasms (×100,000/inhabitants) according to the prevalence of white skin color. Brazil, 2022. Source: SIH and SIM, adapted by the author.

According to Fig. 3, a significant positive correlation was observed where the states with a higher prevalence of their population belonging to the self-reported white skin color also showed higher rates of morbidity and mortality from skin cancer (expressed per 100,000 inhabitants) in the year 2022.

**Fig. 4:**
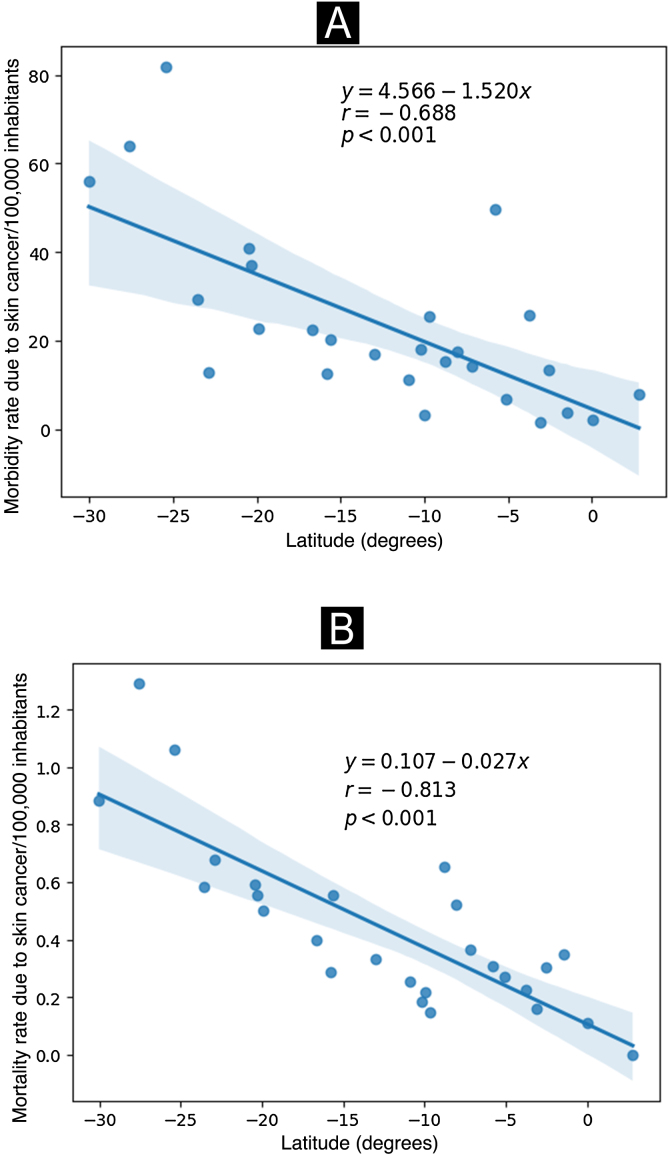
Hospitalization (A) and mortality rates (B) due to malignant skin neoplasms (×100,000/inhabitants) according to the latitude of the Brazilian capitals. Brazil, 2022. Source: SIH and SIM, adapted by the author.

As shown in Fig. 4, a significant negative correlation was observed between the capitals of the Brazilian states of more extreme latitudes with the highest morbidity and mortality rates in the year 2022.

## Discussion

The overall panorama of skin cancer in Brazil is worrisome and showed an increase in its incidence in most federative units in the period from 2012 to 2022, as demonstrated in other studies.^16^, ^17^, ^18^, ^19^, ^20^ Regarding morbidity rates, the southern region stands out, since the three federative units in this region were the ones that stood out regarding the increase in cases of skin cancer in the country. The morbidity rate in the state of Paraná increased from 39.89 to 81.85/100,000 inhabitants, Santa Catarina increased from 21.53 to 63.95/100,000 inhabitants and Rio Grande do Sul from 25.69 to 55.88/100,000 inhabitants. Such occurence in the south of Brazil can be justified by the reduction in the thickness of the ozone layer present in this region, as well as by intermittent and intense sun exposure, less cloud cover, greater longevity of the population, and greater access to health care leading to a greater number of diagnoses.

According to the Ozone Laboratory of the National Institute for Space Research, for every 10% reduction in the ozone layer, there is a 20% increase in UV-B radiation, which is associated with mutations in human DNA that can develop into non-melanoma skin neoplasms. Moreover, for every 1% reduction in this layer, there is a 2% increase in cases of skin cancer.^21^, ^22^ From this perspective, it is important to note that skin tumors, especially non-melanoma skin tumors, remain both the most common neoplasms among Brazilians and the most common among residents of the southern region of Brazil.^23^

Regarding exposure to UV rays, a meta-analysis study demonstrated that discontinuous sun exposure increases the likelihood of melanoma by 1.57 times.^24^ Another study also found that intermittent sun exposure increases the risk of cutaneous melanoma by 61%, while chronic sun exposure does not increase the risk of this neoplasm to the same extent.^25^ Hence, the south of Brazil is the main region exposed to sunlight on an intermittent basis, due to the fact that this region presents harsher winters when compared to the rest of the country and consequently sun exposure occurs usually only during the summer.

Also in relation to the southern region, a study that compared geographic and social inequalities in access to health services in Brazil showed that the population living in the most developed regions (southern and southeastern) demonstrated higher rates of health service use.^26^ In this regard, the southern population has more resources for the screening of skin lesions, which leads to a greater number of appropriate diagnoses. Moreover, according to Baltes and Smith, comorbidities intensify from the fourth decade onwards, favoring the appearance of various types of neoplasms.^27^ It is known that developed regions have more elderly individuals, thus allowing the emergence of skin cancer.

Given that the tropical zone has the highest incidence of sunlight on the planet, sun exposure among Brazilians is higher when compared to countries in the temperate and polar zones. Considering the territory of Brazil, the total annual sunshine is more pronounced in the northeastern, southeastern and midwestern regions.^28^ From this perspective, in the present study, the states of Espírito Santo, Minas Gerais, Mato Grosso do Sul, Alagoas, Rio Grande do Norte and Ceará also showed considerable increase in morbidity rates, corroborating the fact that sun exposure is very important for the development of skin neoplasms.

The federative units that showed stability regarding the morbidity rate belong, in their majority, to the northern region of the country. Moreover, the incidence of skin cancer in these states (Acre, Amazonas, Roraima, Amapá and Pernambuco) is minimal when compared to the other states. Therefore, it is recapitulated that the evolution of skin neoplasms has several risk factors, among them the population ethnicity.^6^ According to data from the Brazilian Institute of Geography and Statistics (IBGE), the north of Brazil is predominantly brown (69.2%), and concentrates the majority of the indigenous population in the country with the lowest predominance of white population in Brazil.^11^ Thus, considering the Fitzpatrick classification, individuals who belong to phototypes III, IV and V are less predisposed to developing skin tumors and have a family history of low risk for this type of cancer.^6^

Regarding skin cancer mortality rates, the mortality rate per/100,000 inhabitants due to malignant skin neoplasms in the southern states is significantly higher than in any other state in Brazil.^29^ The study in question evaluated data related to skin cancer from 2012 to 2022, and the states of Santa Catarina, Paraná and Rio Grande do Sul continue to stand out in relation to deaths due to these neoplasms in 2022. Similar to what was observed in São Paulo and Minas Gerais, which also had significantly increased mortality rates during this period, thus justifying the predominance of light-skinned individuals in these regions (southern and southeastern).

The immigration of people from Central Europe to Brazil was more pronounced in the southern and southeastern regions, where, for geographical reasons, they experienced little racial mixing and therefore lighter skin phototypes prevailed, which is a risk factor for the development of malignant skin lesions.^11^

Additionally, a significant portion of the European immigrants who settled in Brazil, as well as their descendants, worked in agricultural activities, facing constant exposure to solar radiation. Thus, individuals with white skin and continuous exposure to UV rays, constitute the main phenotype for death due to malignant skin neoplasia.^30^

However, although the southern region stands out in skin cancer mortality, Brazil showed an increase in the mortality rate from 0.35 to 0.54/100,000 inhabitants. According to the National Cancer Institute (INCA, *Instituto Nacional de Câncer*), this is due to the combination of more adequately performed diagnoses and an increase in the population life expectancy, given that the majority of patients who die of skin cancer are elderly individuals.^8^

The scenario of patients affected by skin cancer in Brazil between 2012 and 2022 consisted predominantly of male individuals, with hospitalization rates of 63.8/100,000 inhabitants and mortality rates of 58.9/100,000 inhabitants. According to a study conducted by Ciążyńska et al.,^31^ there is a higher prevalence of skin cancer among men compared to women. This can be attributed, in part, to the fact that men used to be more exposed to sunlight in outdoor work and sports activities. However, it is important to note that there have been recent changes in attitudes towards recreational sun exposure, resulting in a specific increase in exposure to ultraviolet radiation (UVR) in people of all genders from an early age.^31^

In addition, in the Brazilian context, significant cultural transformations have occurred, mainly since the 1920s, which have further intensified outdoor leisure activities, resulting in excessive exposure to ultraviolet solar radiation, especially among young people. At the same time, one continues to observe work activities that involve sun exposure throughout the national territory, as is the case of rural workers and informal commerce on the streets of urban centers.^32^ A study carried out in Pernambuco, which included fishermen, revealed a high prevalence of lesions caused by sun exposure.^33^

International studies^24^, ^34^ corroborate the association between exposure to ultraviolet (UV) radiation and the development of melanoma. A study conducted in France found that 83% of the 10,340 cases of melanoma recorded in 2015 were attributed to UV exposure.^34^

Regarding age, it is observed that morbidity and mortality rates are higher in people over 50 years of age, with a particularly high morbidity rate in patients over 80 years of age. This is due to photocarcinogenesis, which is caused by continuous sun exposure and whose cumulative effect justifies the increased risk of skin cancer in older individuals. This suggests that the more advanced the age, the greater the risk of developing these neoplasms.^12^, ^35^ Moreover, according to a time series study,^36^ there has been an increase in hospitalization rates of elderly people due to neoplasms in the last decade, which can also be explained by population aging and the epidemiological transition, resulting from the decline in hospitalizations due to infectious diseases and the increase in chronic non-communicable diseases (CNDs).

According to a North American analysis of a 30 years period of skin cancer screening in the United States, the diagnosis of melanoma, the type of cancer most frequently found in scientific studies due to its higher lethality,^37^ doubled in individuals with a mean age of 60 years or older.^38^ However, the present study did not differentiate between the types of malignant skin neoplasms.

Regarding self-reported skin color, white skin colored people presented the highest morbidity and mortality rates. As already explained, people who have phenotypic markers of susceptibility to UV radiation, such as fair skin, freckles, light eyes and hair, and inability to tan, have an increased risk of skin cancer.^39^

The frequency of malignant skin neoplasms is recognized as significantly lower in individuals with darker skin when compared to those with white skin. However, although the occurrence is lower among the former, it is often associated with unfavorable prognoses. The term “individuals with darker skin” encompasses people of African, Asian, Native American, Middle Eastern and Hispanic origins. Generally, these individuals have more pronounced pigmentation, specifically classified as Fitzpatrick types III and IV.^40^

Regarding the latitude of the Brazilian states, a negative correlation was observed, that is, as the latitude decreases, the morbidity and mortality rates due to malignant skin neoplasms increase. This trend can be explained in part by the process of miscegenation that occurred in the southern and southeastern regions of Brazil.^41^ Australia, a country at a lower latitude that was largely settled by people of Caucasian descent, and nowadays has approximately 95% of its population of European or Anglo-Saxon descent, has the highest incidence of skin cancer in the world.^41^

Furthermore, according to Crombie's research, the connection between melanoma and latitude may not be observed in the extreme north, since both Finland and Iceland have lower incidence rates of melanoma than Norway and Sweden, which are located immediately to the south. This would suggest that across Europe there is a range of skin tones, ranging from darker tones in the south to lighter ones in the north, resulting in different levels of susceptibility to melanoma induced by UV exposure. The influence of this susceptibility is significant enough to outweigh the opposite effect of reduced UV intensity at higher latitudes, thus highlighting the risks of excessive sun exposure for fair-skinned individuals.^42^

Among the study limitations, the characterization of skin color stands out, which was self-reported and may not be completely aligned with phototypes. Additionally, it is necessary to address sample selection bias, since the analyzed data are exclusively from hospitalizations through the Brazilian Unified Health System (SUS, *Sistema Único de Saúde).* Also, while the predominant treatment for skin cancer is carried out on an outpatient basis, the study focused on hospitalizations and mortality, which reflects the most severe cases of the disease.

Moreover, the ecological design addresses important issues from the collected secondary data. These limitations may be the underreporting of secondary data and in-cluster data routes. Secondary data are often subject to underreporting or recording errors. This can arise from several reasons, including inconsistent data collection practices, underreporting of events, or changes in reporting policies over time. These limitations may influence the accuracy of the information used in the analysis.

An ecological study involves an analysis of data at the group or cluster level, rather than individual data. This method can lead to “cluster ecology” and make it difficult to attribute results to individuals within groups. Therefore, it is important to consider that the conclusions drawn from the study are based on associations observed at the group level and cannot be directly extrapolated to the individual level. For this reason, it is difficult to draw causal links from ecological explorations.

## Conclusion

The results obtained in relation to morbidity and mortality due to skin neoplasia during the assessed period indicated an increase in most states. Among the hypotheses one can list: the thinner ozone layer, less cloud cover, population aging, internal migrations and greater access to diagnosis. The states in the southern region showed higher rates, which can be explained by the predominance of individuals with light skin, genetics and occupational exposure to the sun. The states of Espírito Santo, Minas Gerais, Mato Grosso do Sul, Alagoas, Rio Grande do Norte and Ceará showed a significant increase in morbidity rates, confirming that the greater annual total solar insolation exposes their populations to the risk of developing skin neoplasias. On the other hand, the stability in morbidity observed in states in the north region, such as Acre, Amazonas, Roraima, Amapá and Pernambuco, deserves to be highlighted, since it confirms the influence of the population ethnicity.

Regarding morbidity and mortality by gender, the highest rate was observed in the males. This can be attributed to the greater exposure of men to outdoor environments and sports activities. Regarding the self-reported skin color variable, white-skinned individuals had higher rates of morbidity and mortality, due to the greater predisposition of this population to the development of apparent neoplasms. Moreover, morbidity was higher among people over 50 years of age, while the highest mortality rate was observed among people over 80 years of age. This age group is especially susceptible to carcinogenesis due to continuous exposure to the sun, whose cumulative effect increases the risk of skin cancer, as well as factors such as population aging and epidemiological transition.

Furthermore, an inverse panorama is observed between latitude and morbidity and mortality due to malignant skin neoplasms, that is, as latitude increases, morbidity and mortality rates decrease. This trend can be attributed, in part, to the influence of European immigration in the southern and southeastern regions of the country, where the majority of the population has a lighter skin color.

This study reinforces the importance of considering sun exposure, age group, gender, ethnic and genetic diversity in the Brazilian population when developing strategies for skin cancer prevention and awareness. Therefore, public health policies must be sensitive to these nuances, promote early detection and ensure access to adequate health services to fight this alarming trend of increasing skin cancer in the country.

## Financial support

None declared.

## Authors’ contributions

Lucía Alejandra Bolis Castro: Data collection; analysis and interpretation of data; drafting and editing of the manuscript; critical review of the literature; approval of the final version of the manuscript.

Maria Cecília Aires Martins: Data collection; analysis and interpretation of data; drafting and editing of the manuscript; critical review of the literature; approval of the final version of the manuscript.

Kelser de Souza Kock: Design and planning of the study; analysis and interpretation of data; statistical analysis; critical review of the literature; approval of the final version of the manuscript.

## Conflicts of interest

None declared.
